# Evaluation of structured and seed-blend refuges for corn earworm (Lepidoptera: Noctuidae) using emergence cages and screen-bagged ear methods

**DOI:** 10.1093/jisesa/ieag030

**Published:** 2026-04-05

**Authors:** Suhas S Vyavhare, G David Buntin, Joni L Blount, Matthew Carroll, Pat Porter

**Affiliations:** Department of Entomology, Texas A&M AgriLife Research and Extension Center, Lubbock, TX, USA; Department of Entomology, University of Georgia, Griffin, GA, USA; Bayer CropScience, Creve Coeur, MO, USA; Bayer CropScience, Creve Coeur, MO, USA; Department of Entomology, Texas A&M AgriLife Research and Extension Center, Lubbock, TX, USA

**Keywords:** refuge strategy, moths, survival, resistance management

## Abstract

The corn earworm, *Helicoverpa zea* (Boddie), is a major lepidopteran pest of corn (*Zea mays* L.) in the United States and is widely managed using transgenic corn hybrids expressing *Bacillus thuringiensis* (Berliner) (Bt) proteins. Seed-blend refuges, in which Bt and non-Bt seeds are mixed in fixed proportions, have been widely adopted as a convenient insect resistance management (IRM) strategy. However, concerns remain about their efficiency in producing susceptible moths compared to a structured block refuge. To evaluate this, five field experiments were conducted across Texas and Georgia during 2020 to 2021 to compare adult *H. zea* emergence from non-Bt refuges configured as structured 100% non-Bt blocks or as 80:20 Bt/non-Bt seed blends. Adult production was quantified using two approaches: emergence cages that captured pupal-to-adult survival and screen bags that confined larvae on individual ears. Both methods yielded comparable estimates of adult *H. zea* emergence. Across all locations and years, seed-blend plots consistently produced fewer moths than expected based on the 20% refuge proportion, yielding 61% to 91% of the adults produced from structured refuges. Screen bag assays similarly showed reduced larval and pupal recovery from seed-blend ears. Non-Bt ears from seed-blend plots did not consistently differ from structured refuges in larval feeding intensity. Findings from this study indicate that seed-blend refuges may underperform in producing susceptible moths and may not consistently function as an effective standalone refuge strategy.

## Introduction

The corn earworm, *Helicoverpa zea* (Boddie), is a significant pest affecting several field crops, including cotton (*Gossypium hirsutum* L.), soybean (*Glycine max* L. Merrill), sorghum (*Sorghum bicolor* L. Moench), and corn (*Zea mays* L.). While *H. zea* occurs on many cultivated crops and non-cultivated wild hosts, corn is its preferred host (Johnson et al. 1975). In field corn, *H. zea* is typically not considered an economic pest, although in late-planted corn may cause moderate yield loss in the southern U.S. (Buntin 2008, Reay-Jones et al. 2014, 2025, Reay-Jones 2019). However, in certain years, infestations can negatively impact seed quality by facilitating fungal colonization and enhancing mycotoxin production in host tissues. Infestations of *H. zea* are positively correlated with seed contamination by fumonisins produced by *Fusarium verticillioides* strains (Chalivendra et al. 2020, Barton et al. 2024).

The management of *H. zea* in corn heavily relies on the use of plant-incorporated *Bacillus thuringiensis* Berliner (Bt) traits. Since the commercialization of Bt crops in the U.S. in 1996, these traits have proven highly effective, with over 85% of corn acreage in the country planted with varieties expressing Bt proteins in 2023 (USDA ERS 2024). The benefits of Bt technology include reduced chemical insecticide use, conservation of beneficial insects, and increased crop yields (National Research Council (NRC) 2010). However, rapid adaptation to Bt proteins in pest populations presents a significant challenge, as resistance in field populations of insects has become widespread. Resistance to Cry1Ac, Cry1Ab, Cry1A. 105, and Cry2Ab Bt proteins has been reported in *H. zea* populations in Bt corn or cotton across the U.S. (Dively et al. 2017, Reisig et al. 2018, Yang et al. 2018, 2019, Bilbo et al. 2019, Kaur et al. 2019, Kerns et al. 2019, Little et al. 2019).

To preserve the efficacy of Bt proteins and delay the development of resistance in insect populations, the planting of refuges is a widely recommended strategy (Tabashnik et al. 2013). Structured refuge involves planting blocks of non-Bt crops adjacent to Bt crops, while seed blend refuge involves planting a mixture of Bt and non-Bt seeds at specific proportions (eg 90:10, 95:5) (Tabashnik et al. 2008). The theory behind refuges is that non-Bt host plants will produce susceptible insects, which will then mate with the few resistant individuals emerging from Bt plants (Carrière et al. 2010, Tabashnik et al. 2013). Refuges are particularly effective in delaying resistance when it is inherited as a recessive trait, as heterozygous individuals do not survive on Bt plants. However, if resistance is inherited as a dominant trait, heterozygous offspring produced by the mating of resistant and susceptible individuals can survive on Bt crops, potentially undermining the effectiveness of refuge in delaying resistance.

Seed-blend refuges are the most commonly used refuge type in the U.S. Corn Belt due to their ease of implementation. They are also permitted in cotton-producing regions of the southern U.S. but must be accompanied by the appropriate amount of structured refuge. Seed blends alone do not fulfill insect resistance management (IRM) requirements in cotton-growing regions because cotton also expresses Bt toxins and *H. zea* is a shared pest of both crops. The first two generations of *H. zea* typically develop on corn, followed by a generation developing on cotton (Reay-Jones 2019). Because there is no refuge requirement in cotton, the refuge in corn within the cotton growing regions is increased relative to that in the Corn Belt.

The seed-blend refuge strategy, although convenient for growers to meet federal IRM regulations, presents several potential risks. Larval movement between Bt and non-Bt plants within seed blends can accelerate resistance evolution by increasing the functional dominance of resistance or by reducing the survival of susceptible insects, thereby diminishing the effective size of refuges (Mallet and Porter 1992). For example, Brévault et al. (2015) demonstrated that the dominance of resistance to Cry1Ac in *H. zea* was significantly higher in a seed mixture than in a structured block of Bt cotton. Movement of larvae from refuge plants to Bt plants can reduce the production of susceptible adults, weakening the effectiveness of the refuge strategy (Head and Greenplate 2012, Yang et al. 2020, Guo et al. 2021, Vyavhare et al. 2021).

In addition, cross-pollination in corn can result in Bt toxin expression in kernels of refuge plants within seed blends. Because Bt traits segregate in pollen from Bt plants, individual kernels on non-Bt ears that receive Bt pollen may express one or more Bt toxins. Exposure of larvae to these kernels could increase mortality of susceptible individuals, promote survival of heterozygotes, and enhance selection for resistance (Yang et al. 2014, Vyavhare et al. 2021). Such effects could further reduce the effective refuge size and compromise the intended resistance management benefits of the refuge strategy.

Despite these concerns, studies evaluating *H. zea* adult emergence from structured and seed-blended refuges have produced inconsistent results. Towles et al. (2021) reported no significant differences in *H. zea* adult emergence between the two refuge strategies, whereas Vyavhare et al. (2021) observed significantly fewer adults emerging from seed-blend refuge ears compared to structured refuge ears in 2 of 3 yr. Similarly, Guo et al. (2021) reported a 70% reduction in pupal and adult production from seed-blend refuge ears relative to structured refuges, along with lighter pupae. In contrast, Yang et al. (2014) found that Bt pollen contamination did not significantly affect early larval survival but delayed larval development by approximately one instar. These conflicting findings highlight the need for additional field-based evaluations to better quantify the effectiveness of seed-blend refuges in producing susceptible *H. zea* adults.

The primary objective of this study was to assess the efficiency of seed blend refuges in producing *H. zea* moths relative to non-Bt structured refuges. A second objective was to compare the use of the two methods, emergence cages and screen bagged ears for assessing *H. zea* production from non-Bt ears. Understanding the efficacy of seed blend refuges is crucial to determining whether they can produce the expected number of adults to delay the development of Bt resistance in field populations of *H. zea*.

## Materials and Methods

Five field experiments were conducted across three locations in Texas and Georgia during the 2020 and 2021 growing seasons. The locations included commercial farms in Olton, Texas (2020 and 2021), Lubbock, Texas (2021), and Griffin, Georgia (2020 and 2021). The common experimental design used for all years in all locations was a randomized complete block (RCB) with three treatments and four replications. The treatments were 100% non-Bt corn, 80% Bt hybrid with 20% non-Bt seed blend, and 100% Bt hybrid. The field corn hybrids used in the study were Dekalb DKC 66-94 Roundup Ready 2 (non-Bt) and DKC 66-29 Trecepta corn (Bt traits: Cry1A.105/Cry2Ab2/Vip3Aa20) (Bayer CropScience, St. Louis, MO). The research plots were maintained according to local agronomic recommendations for field corn. The field in Olton was irrigated using a central pivot system, while the field in Lubbock was irrigated with a subsurface drip system. Plots were irrigated using lateral overhead irrigation system in Georgia as needed to prevent moisture stress. Corn was planted on 0.76 m row centers at a seeding rate of 86,114 seeds per hectare. The plots measured 16 rows by 30.48 m in Texas and 16 rows by 15.24 m in Georgia.

*H. zea* infestation levels were monitored by inspecting non-Bt ears from both the 80:20 seed blend and 100% non-Bt plots during the milk stage of corn. In seed blend plots, all non-Bt ears from the selected rows in the center of the plot were carefully inspected by slightly opening the husk at the ear tip without damaging the ear or dislodging any larvae. Plants identified as non-Bt based on larval presence were marked with flagging tape, and a subset of these plants (6 per plot) was verified as non-Bt using EnviroLogix QuickStix (EnviroLogix Inc., Portland, ME) that detect Vip3Aa20 protein. Two types of cages were used to capture *H. zea* adults emerging from each plot: (i) aluminum screen bags to hold developing larvae on ears until adulthood and (ii) emergence cages placed over soil to determine the number of adults emerging from soil after pupation. The experiment in Lubbock, TX in 2021 utilized only screen bags for adult emergence capture.

### Emergence Cages

The design of the emergence cages used in this study was similar to that described by Towles et al. (2021). The cages were pyramid-shaped, measuring 1.321 m in length and 0.762 m in width, covering an area of 1.0 m^2^. Trap frames were constructed from 3.2 mm steel rods and covered with 6.4 mm mesh galvanized aluminum screen. An exit hole for moths was created at the top of each cage using a metal tube with a diameter of 2.5 cm. To capture the moths, plastic 473 ml (16 oz) serving cups with matching lids were affixed to the exit holes outside the cages. Emergence cages were installed on the ground after larvae vacated the corn ears to pupate in, the soil, but before moths began to emerge. Corn plants inside emergence cages were removed by cutting the stalks approximately 10 cm above the soil level using a gasoline-powered brush cutter in all rows designated for the emergence cages. Cut stalks were arranged around the edges of the cages to secure them in place and minimize movement of larvae and moths out of the cages.

#### Texas

In 2020, emergence cages were deployed in rows 7 to 10 of the 16-row plots on August 5 and held in place until September 4. The cages were installed to ensure at least one plant within each cage exhibited signs of active *H. zea* infestation as determined by ear inspection. Two adjacent rows were examined for ear infestations, but only one row was covered by a cage. In 2021, cages were deployed on July 30 and held in place until September 17 in rows 7 to 9 without the requirement of having evidence of infestation on at least one plant within the cage. There was an average of 7.7 cut corn stalks per cage in 2020 across all replications. These data were not recorded in 2021. Each year, there were 8 cages per plot in the 100% non-Bt and 100% Bt hybrid plots, and 14 cages per plot in the 80:20 seed blend plots. Due to limited availability, fewer cages were installed in the 100% non-Bt and 100% Bt plots. Adult emergence of *H. zea* was recorded a minimum of twice a week until no moths were observed for seven consecutive days.

#### Georgia

Emergence cages were installed on July 30 and August 5 in 2020; and 2021; respectively, in rows 9 to 12. The center rows of the 80:20 seed blend plots were inspected for signs of *H. zea* infestation, and the row with the closest ratio of refuge plants to 20% was selected for caging. Cages were placed end-to-end to cover all cut plants in the selected row. The number of cages varied slightly in 2020; some rows required 12 cages while others required 13. All plots in 2021 contained 13 cages per plot. The number of plants per cage averaged 7.76 in 2020 and 7.51 in 2021 and were not significantly different among treatments in either year (2020: *F *= 3.98; df = 2,6; *P *= 0.0793; 2021: *F *= 2.40; df = 2, 6, *P *= 0.1716). In both years, cages were inspected every other day for 58 d (2 wk after no new moths had emerged). *Ear damage:* Assessment of ear tip and kernel area damage caused by *H. zea* was conducted at the R6 stage of corn by inspecting 50 ears in the 100% non-Bt and 100% Bt hybrid plots, as well as one entire row of plants in the 80:20 seed blend plots. Damage measurements were done in a row without bagged ears of emergence cages and not adjacent to the rows with emergence cages.

### Screen Bags

Screen bags (38 × 15 cm, made of aluminum window screening) were installed only on non-Bt ears from the 80:20 seed blend and 100% non-Bt plots that exhibited signs of active larval feeding. In the 100% Bt hybrid plots, bags were installed on randomly selected ears because no ears exhibited signs of larval feeding. Ear feeding prior to bag installation was assessed by gently pulling back the silk until silk clipping or fresh frass was observed. Minimal manipulation of the ears was practiced, and, after assessment, ears were resealed and flagged for subsequent screen bag installation. All screen bags were installed on the same day when the majority of ears had larvae nearing pupation. Screen bags were sealed at the base of the ear using a steel worm gear hose clamp.

In Texas, rows 5 and 6 of each plot were designated for bagged ears. Eight bags per plot were installed in the 100% Bt hybrid plots, while 14 bags per plot were installed in the 100% non-Bt and 80:20 seed blend plots. Fewer screen bags were installed in the 100% Bt plots due to limited availability and the expectation, based on prior experience, that Vip3Aa20 corn would produce little to no *H. zea.* In Olton, the screen bags remained on ears for 34 and 26 d after installation in, 2020; and 2021; respectively. In Lubbock, the bags remained for 21 d after installation. In Georgia, rows 5 through 7 were used for bagged ears, with bags installed on 15 ears per plot. Screen cages were installed on July 27 and July 23 in 2020; and 2021; respectively. The bags remained on the ears for 31 d in 2020 and 32 d in 2021. At the termination of ear confinement in screen bags, each plant was cut below and above the ear, leaving the bag attached to a short section of the stalk. These units were transported to the laboratory and dissected to count the number of dead and alive *H. zea* larvae, pupae, and adults. Data on the extent of ear tip and kernel feeding by *H. zea* were recorded for both, 2020; and 2021; in Georgia.

### Data Analysis

Data were analyzed for each experiment separately. Cumulative total *H. zea* emergence from emergence cages was calculated for each plot across all sample dates and converted to moths per hectare. Data were analyzed to compare *H. zea* emergence across three treatments. Percent optimal *H. zea* recovery in seed blend plots was determined by comparing moth emergence from 80:20 seed blend to that of 20% from 100% non-Bt plots. Data on *H. zea* emergence and amount of kernel and ear tip feeding by *H. zea* were analyzed separately for each experiment with one way analysis of variance (ANOVA) using the R statistical computing software. Bt corn treatments were considered fixed effects and replication as a random effect. Means were separated by Tukey’s HSD procedures at the α = 0.05. For *t*-test: GraphPad Prism software (version 10.6.1, GraphPad Software, San Diego, California, United States) was used.

## Results

### Emergence Cage Experiments

**Fig. 1.**
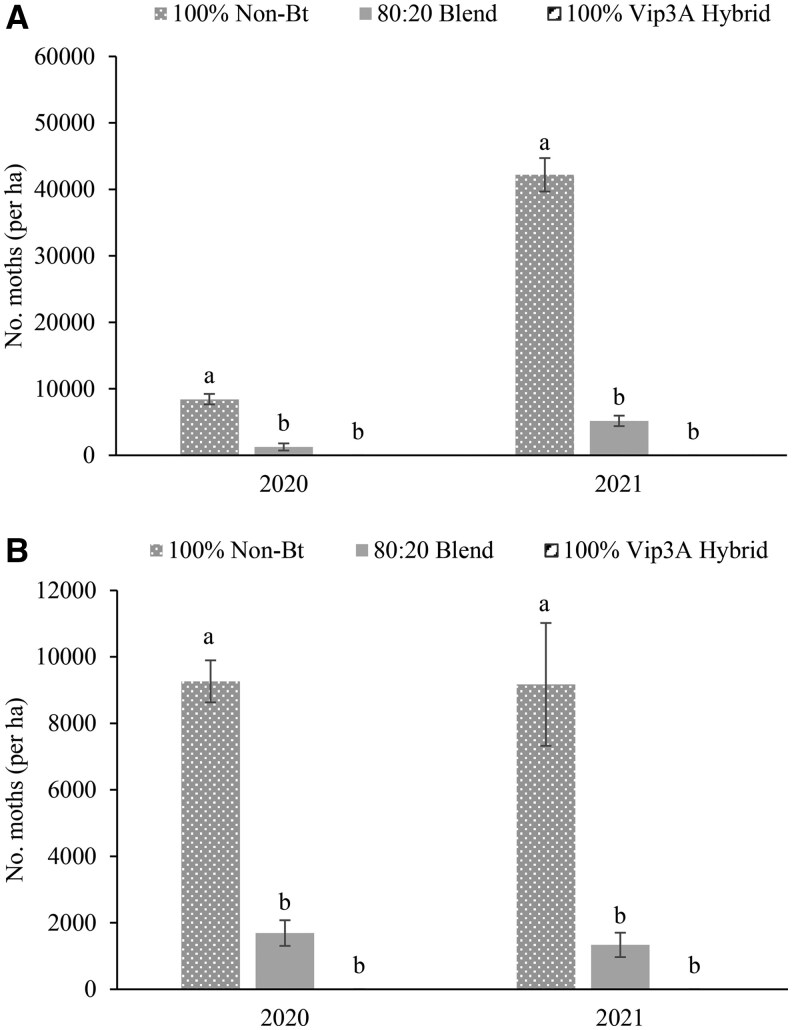
Mean *H. zea* moths captured per hectare in emergence cages in Texas A) and Georgia B) in 2020 and 2021. Means within location and year with the same letter are not significantly different (alpha = 0.05).

The mean number of adult moths captured from emergence cages varied significantly across treatments in both 2020 and 2021 in Texas (2020: *F *= 73.21, df = 2, 6; *P *= 0.000061; 2021: *F *= 216.71, df = 2, 6; *P *= 0.000003) (Fig. 1A) and Georgia (2020: *F* = 95.34, df = 2, 6; *P *= 0.000028; 2021: *F* = 24.11, df = 2, 6; *P *= 0.0013) (Fig. 1B). No *H. zea* moths were captured from the emergence cages in 100% Bt hybrid plots. The number of moths recovered was significantly greater in the non-Bt hybrid than the seed blend plots in all four experiments. In Texas, the percent optimal emergence from 80:20 seed blend plots (ie 20% of emergence from emergence values in, the 100% non-Bt plots) was 74% and 61% in, 2020; and 2021; respectively. In Georgia, the percent optimal emergence from 80:20 seed blend was 91% and 73% in 2020; and 2021; respectively.

**Fig. 2.**
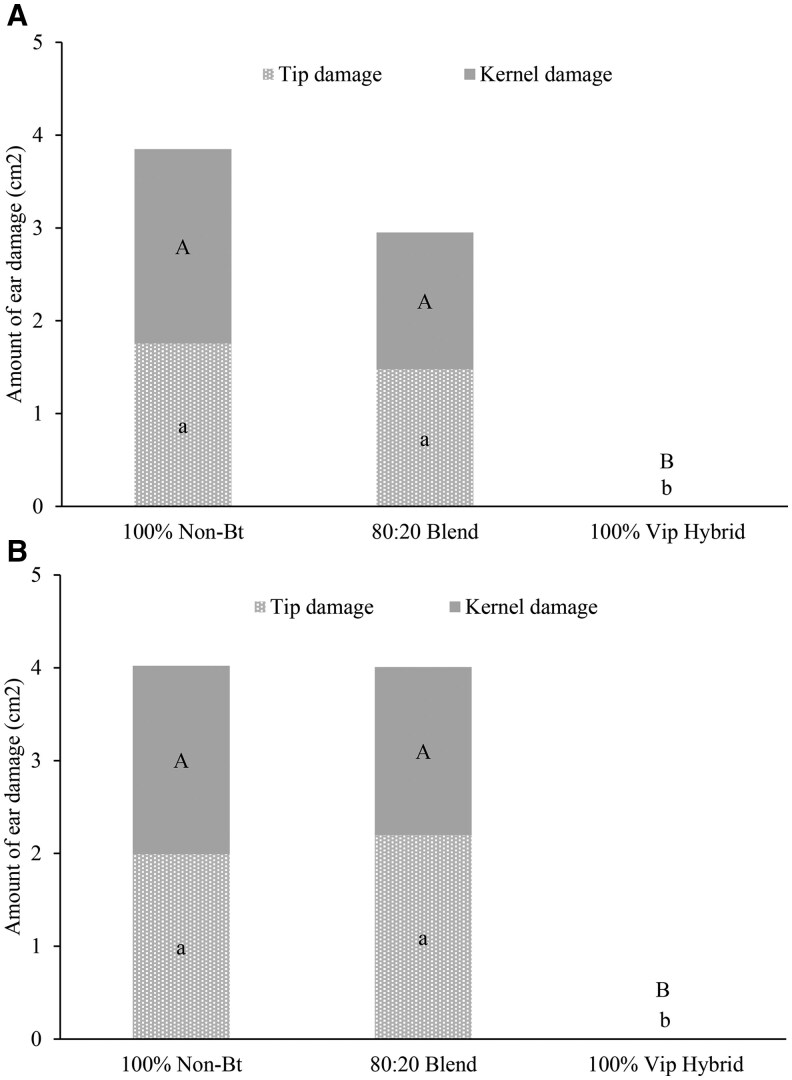
Amount of tip and kernel damage to infested ears from non-caged plants in a row adjacent to rows with emergence cages, Georgia 2020 A) and 2021 B). Bars with the same uppercase letter are not significantly different for kernel damage, and bars with the same lowercase letter are not significantly different for tip damage (alpha = 0.05).

Although the differences between amount of ear tip (2020: *F* = 73.20, df = 2, 6; *P *= 0.0001; 2021: *F* = 177.24, df = 2, 6; *P *= 0.0001) and kernel (2020: *F* = 22.99, df = 2, 6; *P *= 0.0003; 2021: *F* = 177.53, df = 2, 6; *P *= 0.0001) damage from *H. zea* were significant across three treatments, no significant differences were observed for ear damage between non-Bt ears of 80:20 seed blend and 100% non-Bt plots (Fig. 2).

### Screen Bag Experiments

**Fig. 3.**
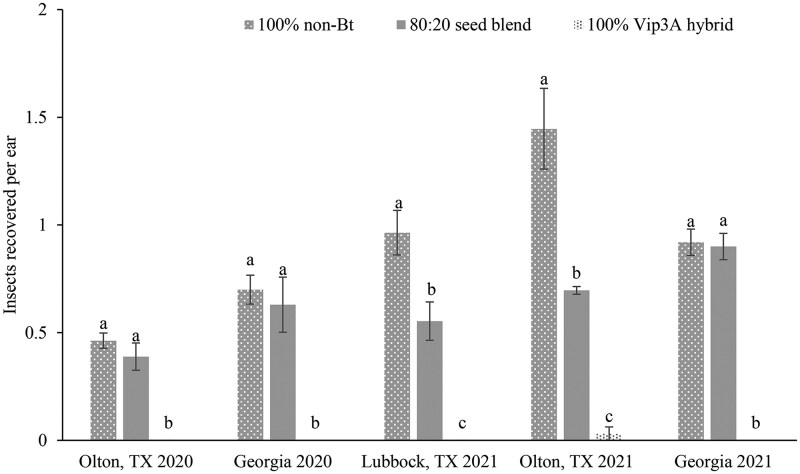
Mean number of 6th instar larvae (dead) with exit holes, pupae or prepupae, or adult *H. zea* moths recovered from screen bagged ears of corn in five experiments in Texas and Georgia. Bars with the same letter within each location and year are not significantly different (alpha = 0.05).

**Fig. 4.**
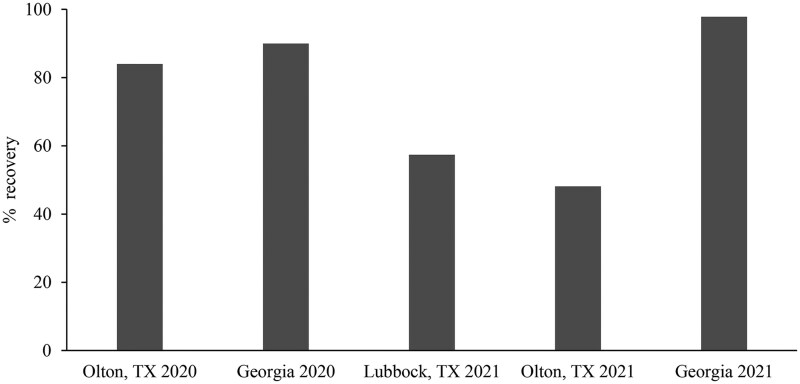
Percent recovery of *H. zea* from 80:20 seed-blend refuge ears compared with 100% non-Bt ears collected from screen bags across experiments.

The mean density of *H. zea* per bagged ear varied significantly among treatments in Texas (Olton, TX, 2020: *F* = 34.53, df = 2, 6, *P *= 0.00051; Lubbock, TX 2021: *F* = 35.72, df = 2, 6, *P *= 0.00046; Olton, TX 2021: *F* = 46.06, df = 2, 6, *P *= 0.00022) and Georgia (2020: *F *= 63.65, df = 2, 6; *P *= 0.000091; 2021: *F* = 78.88, df = 2, 6, *P *= 0.000049) (Fig. 3). No significant differences were observed in mean density of *H. zea* between ears of 100% non-Bt and refuge ears in 80:20 seed blend plots in both years in GA and in 2020 in Texas. Mean density of *H. zea* was significantly lower in seed-blend ears compared to block refuge ears in both 2021 experiments in Texas. In 2021, one *H. zea* pupa was found in a bagged ear from the 100% Bt plot in Olton. No *H. zea* were found in bagged ears from 100% Bt plots in the other experiments. The percent recovery of *H. zea* from 80:20 seed blend refuge ears compared to block refuge ears ranged from 48% to 84% in Texas (Fig. 4). In Georgia, it ranged from 90% to 98% between 2 yr.

**Fig. 5.**
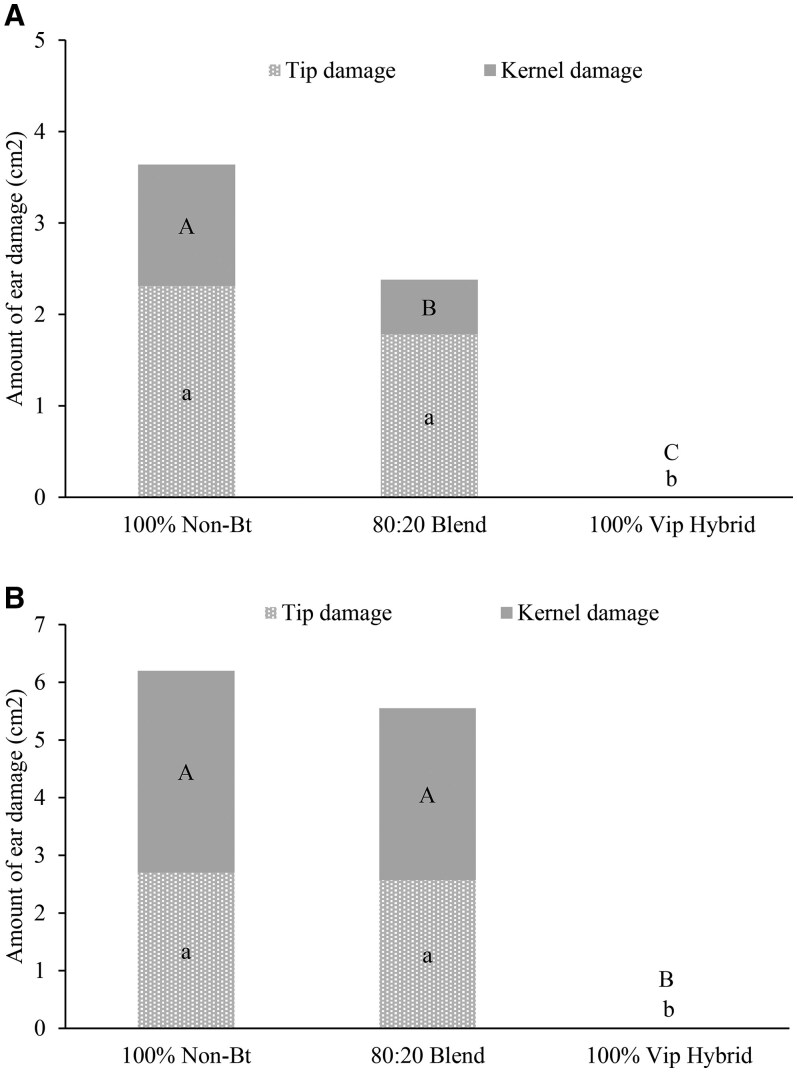
Mean tip and kernel area (cm^2^) damaged by *H. zea* of individual caged (bagged) ears of corn in Georgia in 2020 A) and 2021 B). Bars with the same uppercase letter do not differ significantly for kernel damage, and bars with the same lowercase letter are not significantly different for tip damage (alpha = 0.05).

In Georgia, significant differences were observed in the amount of *H. zea* damage to ear tips (2020: *F* = 23.28, df =2, 6; *P *= 0.0015; 2021: *F* = 112.33, df = 2, 6, *P *= 0.0001) and kernels (2020: *F* = 17.66, df = 2, 6, *P *= 0.0031; 2021: *F* = 4.78, df = 2, 6, *P *= 0.0365) among three treatments (Fig. 5). However, there were no significant differences in amount of tip damage between ears from 80:20 seed blend and 100% non-Bt refuge plots. Amount of damage to kernels varied significantly between 80:20 seed blend and 100% non-Bt refuge plots in, 2020; but not in, 2021; In 2020, a significantly lower amount of *H. zea* damage to kernels was observed in 80:20 seed blend ears compared to ears from 100% non-Bt plots. No injury from *H. zea* was noticed in ears from 100% Bt plots across all experiments.

### Comparison of Moth Recovery of Bagged Ear Method with the Emergence Cage Method

Averaged over the four experiments the percent recovery was 75.80 ± 7.67% for emergence cages and 80.18 ± 11.04% for bagged ear cages which was not statistically significantly different (*t* = 0.3254, df = 6, *P* = 0.7559). This suggests that the two cage methods provided comparable results when measuring*H. zea* emergence from blended corn. The recovery of *H. zea* from screen bagged ear cages in Lubbock, TX was 57% in 2021 but was not included in the average total because no emergence cages were used in this experiment.

## Discussion

Seed-blend refuge is now widely adopted in the U.S. Corn Belt, where blends typically contain 5 to 10% non-Bt seed (DiFonzo 2025). In cotton-producing regions of the southern U.S., current Bt corn registrations require that seed blends be supplemented with an additional structured refuge planted within or adjacent to the seed-blend field (U.S. EPA 2025). Despite this requirement, compliance among growers remains low due to several factors, including unfavorable weather during planting, difficulties in obtaining appropriate refuge seed, planting errors, logistical constraints such as farm layout, limited awareness of refuge importance, lack of incentive to plant non-Bt refuge, and restricted access to high-yielding non-Bt hybrids (ABSTC 2016, Reisig 2017, Reisig and Kurtz 2018).

A survey of corn growers in North Carolina, a major cotton-producing state conducted from 2014 to 2016 found that growers were generally reluctant to plant non-Bt refuge, with only 38.3% to 44.3% reporting plans to do so and an additional 22.0% to 29.4% uncertain about their intentions (Reisig 2017). More recent surveys indicate that only about 10% of producers in the southern region are meeting the additional refuge requirement (U.S. EPA 2022). As a result, seed blends often serve as the primary, and in some cases the only, source of refuge in southern U.S. corn production. The current study demonstrates that seed blends do not always produce the expected number of adult *H. zea*, and in some cases yield fewer moths than structured block refuges.

Although commercially available seed blends commonly contain 5% refuge seed, this study examined a 20% seed blend because this proportion is being considered for Bt corn hybrids in the southern U.S. Using adult emergence from a block refuge as the benchmark, optimal performance of a 20% seed blend would be the production of 20% as many months per hectare as the block refuge. However, emergence cage data indicated that seed blends produced only 74% and 61% of the expected adult moths in Texas in, 2020 and 2021, respectively, and 91% and 73% of the expected adults in Georgia during those same years. Reductions below the expected values were consistent in every year and location. These results indicate that seed blends may not consistently function as intended in producing adults.

The screen-bag experiments further supported these findings. Ears from non-Bt ears in seed-blend refuge plots consistently produced fewer larvae and adults than ears from block refuges in both states, with statistically significant reductions observed in Texas in 2021 (Fig. 3). Because refuge proportion was held constant across years and locations, the reduced emergence is likely attributable to factors other than refuge size such as contamination of refuge ears with Bt pollen from nearby Bt plants. The number of adults produced from refuge plants is not the only factor in evaluating the effectiveness of seed blends; the level of Bt exposure experienced by larvae on seed blend refuge ears is also critical. Refuge ears in seed blends are pollinated by neighboring Bt plants, resulting in kernels that may express one or more Bt toxins. For example, in Texas, 52% to 76% of kernels from non-Bt ears in seed-blend refuges expressed at least one Bt toxin over 3 yr of study (Vyavhare et al. 2021). Larvae feeding on such ears may therefore experience lethal or sublethal exposure, potentially accelerating resistance evolution either by mortality of homozygous susceptible larvae or survival of heterozygous resistant larvae. Toxin expression in non-Bt ears was not quantified during this study. However, feeding damage assessments in Georgia found no consistent differences in ear tip damage from *H. zea* between refuge types but did find significantly less kernel damage in, 2021; and numerically but not statistically significant less kernel damage in, 2020; in the seed blend than in the block refuge. The reduced kernel damage may be due to the mosaic of toxin expression in non-Bt ears within a seed blend. Previous studies have similarly reported reduced *H. zea* populations emerging from seed blends due to Bt protein contamination, along with decreased pupal weight and reduced adult fitness (Burkness and Hutchison 2012, Caprio et al. 2016, Guo et al. 2021, Pezzini et al. 2023). Although the current study did not directly assess developmental effects, the reduced adult emergence observed from seed blends is consistent with earlier findings (Yang et al. 2014, Guo et al. 2021, Vyavhare et al. 2021). Conversely, Towles et al. 2021 using the same emergence cages as in the current study found that *H. zea* moth emergence from blended refuge was similar to the proportion of non-Bt plants in blended corn. Future studies should assess how seed blends influence larval development, adult fitness, and resistance selection under varying larval feeding intensity and environmental conditions such as temperature, drought and soil moisture levels, particularly where Bt expression levels may fluctuate. These data suggest that larval feeding intensity alone does not fully explain the reduced adult emergence observed.

Overall adult moth emergence was lower in Texas than in Georgia, which may reflect differences in environmental conditions, particularly soil moisture and soil texture. Schardong et al. (2024) reported that *H. zea* larvae prefer to pupate in sandy soils and that pupal survival is greatest under intermediate soil moisture levels. In the present study, field experiments in Georgia were conducted under relatively higher soil moisture conditions, with looser, sandier soils, whereas Texas sites were characterized by drier conditions and relatively higher clay content, resulting in more compact soils. These less favorable soil conditions at the Texas sites likely contributed to the reduced adult emergence observed under emergence cages. These environmental factors may influence refuge performance and should be considered in future refuge evaluation studies.

In conclusion, both the screen-bagged ear and emergence cage methods were effective and yielded comparable estimates of *H. zea* adult survival and emergence from pure-stand and blended Bt corn. Given its lower cost and reduced labor requirements, the screen-bagged ear method offers a practical and efficient alternative to emergence cages for future field studies.

Results from this study also indicate that seed-blend refuge does not always produce the expected number of *H. zea* adults and may underperform compared to structured block refuge. These findings should be incorporated into modeling efforts and considered when evaluating the suitability of seed blends for insect resistance management, particularly in regions where seed blends may serve as the primary refuge due to low compliance with block-refuge requirements.

## References

[ieag030-B1] ABSTC 2016. 2015 insect resistance management (IRM) compliance assurance program report for maize borer-protected Bt maize, maize rootworm-protected Bt maize, maize borer/maize rootworm-protected stacked and pyramided maize. MRID 49847001.

[ieag030-B2] Barton WY , Buntin GD, Toews MD. 2024. Bt trait efficacy against corn earworm, *helicoverpa zea*, (lepidoptera: Noctuidae) for preserving grain yield and reducing mycotoxin contamination of field corn. Insects 15:914. 10.3390/insects15120914 39769516 PMC11677160

[ieag030-B3] Bilbo TR , Reay-Jones FP, Reisig DD, et al 2019. Susceptibility of corn earworm (lepidoptera: Noctuidae) to cry1a. 105 and cry2ab2 in North and South Carolina. J. Econ. Entomol. 112:1845–1857. 10.1093/jee/toz062 30924858

[ieag030-B4] Brévault T , Tabashnik BE, Carrière Y. 2015. A seed mixture increases dominance of resistance to bt cotton in *helicoverpa zea*. Sci. Rep. 5:9807. 10.1038/srep09807 25950459 PMC4423431

[ieag030-B5] Buntin GD. 2008. Corn expressing cry1ab or cry1f endotoxin for fall armyworm and corn earworm (lepidoptera: Noctuidae) management in field corn for grain production. Fla. Entomol. 91:523–530. 10.1653/0015-4040-91.4.523

[ieag030-B6] Burkness EC , Hutchison WD. 2012. Bt pollen dispersal and Bt kernel mosaics: integrity of non-bt refugia for lepidopteran resistance management in maize. J. Econ. Entomol. 105:1773–1780. 10.1603/EC12128 23156176

[ieag030-B7] Caprio MA , Martinez JC, Porter PA, et al 2016. The impact of inter-kernel movement in the evolution of resistance to dual-toxin Bt-corn varieties in *helicoverpa zea* (lepidoptera: Noctuidae). J. Econ. Entomol. 109:307–319. 10.1093/jee/tov295 26527792

[ieag030-B8] Carrière Y , Crowder DW, Tabashnik BE. 2010. Evolutionary ecology of insect adaptation to Bt crops. Evol. Appl. 3:561–573. 10.1111/j.1752-4571.2010.00129.x 25567947 PMC3352503

[ieag030-B9] Chalivendra S , Huang F, Busman M, et al 2020. Low aflatoxin levels in *Aspergillus flavus*-resistant maize are correlated with increased corn earworm damage and enhanced seed fumonisin. Front. Plant Sci. 11:565323. 10.3389/fpls.2020.565323 33101334 PMC7546873

[ieag030-B10] DiFonzo C. 2025. The handy Bt trait table for US corn production. https://www.texasinsects.org/uploads/4/9/3/0/49304017/bttraittable_aug2025.pdf

[ieag030-B11] Dively GP , Venugopal PD, Finkenbinder C. 2017. Field-evolved resistance in corn earworm to cry proteins expressed by transgenic sweet corn. PLoS One 12:e0183637. 10.1371/journal.pone.0183637 28036388 PMC5201267

[ieag030-B12] Guo J , Oyediran I, Rice ME, et al 2021. Seed blends of pyramided cry/vip maize reduce *helicoverpa zea* populations from refuge ears. J. Pest Sci. 94:959–968. 10.1007/s10340-020-01307-6

[ieag030-B13] Head GP , Greenplate J. 2012. The design and implementation of insect resistance management programs for Bt crops. GM Crops Food 3:144–153. 10.4161/gmcr.20743 22688689

[ieag030-B14] Johnson MW , Stinner R, Rabb R. 1975. Ovipositional response of *heliothis zea* (boddie) to its major hosts in North Carolina. Environ. Entomol. 4:291–297. 10.1093/ee/4.2.291

[ieag030-B15] Kaur G , Guo J, Brown S, et al 2019. Field-evolved resistance of *helicoverpa zea* (boddie) to transgenic maize expressing pyramided cry1a. 105/cry2ab2 proteins in northeast Louisiana, the United States. J. Invertebr. Pathol. 163:11–20. 10.1016/j.jip.2019.02.007 30825480

[ieag030-B16] Kerns D , Yang F, Gore J, et al 2019. Response of *Helicoverpa zea* to diet-overlay Bt toxins and cotton Bt traits field performance in Texas and the Mid-South. 2019 Beltwide Cotton Conferences; 8–10 January 2019; New Orleans, LA. National Cotton Council of America, Memphis, TN.

[ieag030-B17] Little NS , Elkins BH, Mullen RM, et al 2019. Differences between two populations of bollworm, *helicoverpa zea* (lepidoptera: Noctuidae), with variable measurements of laboratory susceptibilities to Bt toxins exposed to non-Bt and Bt cottons in large field cages. PLoS One 14:e0212567. 10.1371/journal.pone.0212567 30865645 PMC6415783

[ieag030-B18] Mallet J , Porter P. 1992. Preventing insect adaptation to insect-resistant crops: are seed mixtures or refugia the best strategy? Proc. R. Soc. B Biol. Sci. 250:165–169. 10.1098/rspb.1992.0145

[ieag030-B19] National Research Council (NRC). 2010. The Impact of Genetically Engineered Crops on Farm Sustainability in the United States. National Academies Press. 10.17226/12804

[ieag030-B20] Pezzini DT , Reisig DD, Buntin GD, et al 2023. Impact of seed blend and structured maize refuge on *helicoverpa zea* (lepidoptera: Noctuidae) potential phenological resistance development parameters in pupae and adults. Pest Manag. Sci. 79:3493–3503. 10.1002/ps.7529 37139844

[ieag030-B21] Reay-Jones FP , Reisig DD. 2014. Impact of corn earworm injury on yield of transgenic corn producing Bt toxins in the carolinas. J. Econ. Entomol. 107:1101–1109. 10.1603/EC13516 25026670

[ieag030-B22] Reay-Jones FP. 2019. Pest status and management of corn earworm (lepidoptera: Noctuidae) in field corn in the United States. J. Int. Pest Manag. 10:19. 10.1093/jipm/pmz017

[ieag030-B23] Reay-Jones FP , Buntin GD, Reisig DD. 2025. Interactive effects between yields of Bt and non-Bt corn and planting dates in the southeastern United States. J. Econ. Entomol. 118:680–691. 10.1093/jee/toae307 39800387

[ieag030-B24] Reisig DD. 2017. Factors associated with willingness to plant non-Bt maize refuge and suggestions for increasing refuge compliance. J. Int. Pest Manag. 8:9. 10.1093/jipm/pmx002

[ieag030-B25] Reisig DD , Huseth AS, Bacheler JS, et al 2018. Long-term empirical and observational evidence of practical *helicoverpa zea* resistance to cotton with pyramided Bt toxins. J. Econ. Entomol. 111:1824–1833. 10.1093/jee/toy106 29668958

[ieag030-B26] Reisig DD , Kurtz R. 2018. Bt resistance implications for *helicoverpa zea* (lepidoptera: Noctuidae) insecticide resistance management in the United States. Environ. Entomol. 47:1357–1364. 10.1093/ee/nvy142 30277503

[ieag030-B27] Schardong IS , Reisig DD, Possebom T, et al 2024. *Helicoverpa zea* boddie (lepidoptera: Noctuidae) pupal success and adult eclosion across variable soil type and moisture. Environ. Entomol. 53:511–520. 10.1093/ee/nvae045 38778744 PMC11329617

[ieag030-B28] Tabashnik BE , Gassmann AJ, Crowder DW, et al 2008. Insect resistance to Bt crops: evidence versus theory. Nat. Biotechnol. 26:199–202. 10.1038/nbt1382 18259177

[ieag030-B29] Tabashnik BE , Brévault T, Carrière Y. 2013. Insect resistance to Bt crops: Lessons from the first billion acres. Nat. Biotechnol. 31:510–521. 10.1038/nbt.2597 23752438

[ieag030-B30] Towles T , Buntin G, Catchot A, et al 2021. Quantifying the contribution of seed blended refugia in field corn to *helicoverpa zea* (lepidoptera: Noctuidae) populations. J. Econ. Entomol. 114:1771–1778. 10.1093/jee/toab097 34027979

[ieag030-B31] U.S. Department of Agriculture Economic Research Service (USDA ERS). 2024. Recent trends in GE adoption. https://www.ers.usda.gov/data-products/adoption-of-genetically-engineered-crops-in-the-u-s/recent-trends-in-ge-adoption

[ieag030-B32] U.S. Environmental Protection Agency (U.S. EPA). 2022. BPPD review of ABSTC’s 2017—2021 insect resistance managements (IRM) compliance assurance program (CAP) reports for corn borer protected Bt corn, corn rootworm-protected Bt corn, corn borer/corn rootworm-protected stacked and pyramided Bt corn. https://www.regulations.gov/document/EPA-HQ-OPP-2011-0922-0051

[ieag030-B33] U.S. Environmental Protection Agency (U.S. EPA). 2025. Insect resistance management for *Bt* plant-incorporated protectants. https://www.epa.gov/regulation-biotechnology-under-tsca-and-fifra/insect-resistance-management-bt-plant-incorporated

[ieag030-B34] Vyavhare SS , Porter P, Glass S. 2021. Emergence of corn earworm, *helicoverpa zea*, from vip3a seed blend versus structured refuge ears of maize. Southwest. Entomol. 45:853–862. 10.3958/059.045.0402

[ieag030-B35] Yang F , Kerns DL, Head GP, et al 2014. A challenge for the seed mixture refuge strategy in Bt maize: Impact of cross-pollination on an ear-feeding pest, corn earworm. PLoS One 9:e112962. 10.1371/journal.pone.0112962 25409442 PMC4237366

[ieag030-B36] Yang F , Kerns D, Gore J, et al 2018. Continuous monitoring of the susceptibility of Helicoverpa zea in the southern U.S. to different Bt proteins. 2018 Beltwide Cotton Conferences; 3–5 January 2018; San Antonio, TX. National Cotton Council of America, Memphis, TN.

[ieag030-B37] Yang F , González JCS, Williams J, et al 2019. Occurrence and ear damage of *helicoverpa zea* on transgenic bacillus thuringiensis maize in the field in Texas, U.S. and its susceptibility to vip3a protein. Toxins. (Basel) 11:102. 10.3390/toxins11020102 30744120 PMC6416581

[ieag030-B38] Yang F , Kerns DL, Head GP, et al 2020. Extended evaluation of Bt protein cross‐pollination in seed blend plantings on survival, growth, and development of *helicoverpa zea* feeding on refuge ears. Pest Manag. Sci. 76:1011–1019. 10.1002/ps.5611 31498958

